# Physical Activity and Mental Well-being in a Cohort Aged 60–64 Years

**DOI:** 10.1016/j.amepre.2015.03.009

**Published:** 2015-08

**Authors:** Stephanie V. Black, Rachel Cooper, Kathryn R. Martin, Soren Brage, Diana Kuh, Mai Stafford

**Affiliations:** a MRC Unit for Lifelong Health and Ageing at UCL, London; b Epidemiology Group, University of Aberdeen, School of Medicine and Dentistry, Aberdeen; c MRC Epidemiology Unit, University of Cambridge, Institute of Metabolic Science, Addenbrooke’s Hospital, Cambridge, United Kingdom

## Abstract

**Introduction:**

Although evidence suggests physical activity (PA) may be associated with mental well-being at older ages, it is unclear whether some types of PA are more important than others. The purpose of this study is to investigate associations of monitored total PA under free-living conditions, self-reported leisure-time PA (LTPA), and walking for pleasure with mental well-being at age 60–64 years.

**Methods:**

Data on 930 (47%) men and 1,046 (53%) women from the United Kingdom Medical Research Council (MRC) National Survey of Health and Development collected in 2006–2011 at age 60–64 were used in 2013–2014 to test the associations of PA (PA energy expenditure and time spent in different intensities of activity assessed using combined heart rate and acceleration monitors worn for 5 days, self-reported LTPA, and walking for pleasure) with the Warwick-Edinburgh Mental Well-being Scale (WEMWBS; range, 14–70).

**Results:**

In linear regression models adjusted for gender, long-term limiting illness, smoking, employment, socioeconomic position, personality, and prior PA, those who walked for >1 hour/week had mean WEMWBS scores 1.47 (95% CI=0.60, 2.34) points higher than those who reported no walking. Those who participated in LTPA at least five times/month had WEMWBS scores 1.25 (95% CI=0.34, 2.16) points higher than those who did not engage in LTPA. There were no statistically significant associations between free-living PA and WEMWBS scores.

**Conclusions:**

In adults aged 60–64 years, participation in self-selected activities such as LTPA and walking are positively related to mental well-being, whereas total levels of free-living PA are not.

## Introduction

Mental well-being is an essential component of good health^1–6^ and is associated with reduced risk of premature mortality, morbidity, and functional decline.^7–10^ Current interest in positive psychology has led to growing awareness of positive mental well-being, a multidimensional concept capturing hedonic and eudaimonic aspects of mental health and functioning that is distinct from merely the absence of mental disorder.^6^ Among people in their 60s, factors such as retirement and onset of detectable age-related changes in health and function may negatively impact well-being.^11,12^ Therefore, identification of potentially modifiable factors associated with mental well-being at this age is likely to be of public health benefit, especially given increasing governmental awareness of the inevitable health and social challenges associated with global population aging.^13^

Physical activity (PA) is an important modifiable factor that has beneficial influences on physical health across life.^14,15^ Although widely promoted for the benefit of mental well-being, the evidence base primarily relates to the associations between PA and negative mental health.^16,17^ Evidence suggesting beneficial influences of PA on positive mental well-being is also now emerging across all ages, including older adults. Findings from systematic reviews and meta-analyses of intervention studies in healthy older adults have found beneficial effects of participation in short-term exercise programs on aspects of mental well-being, including positive affect,^18–20^ but many studies included in these reviews had small samples. In the few existing observational studies in older adults, self-reported participation in leisure-time PA (LTPA) was also associated cross-sectionally^21–23^ and longitudinally^23^ with better mental well-being.

In the last 20 years, PA measurement has been enhanced by widespread use of portable monitors in population studies to capture free-living PA and relate this to health outcomes.^24,25^ To date, only a few studies have related monitored free-living PA to mental well-being in older adults, finding modest associations.^17,26,27^ A small study of people aged >70 years found weak associations between free-living PA and life satisfaction.^26^ A larger study of people aged ≥65 years found that light-intensity free-living PA was associated with a composite score including life satisfaction, depressed mood, self-rated general health, and social isolation.^27^ Neither study used instruments designed to capture multiple dimensions of hedonic and eudaimonic well-being nor considered the complementary information provided by self-reported PA.^28,29^

Monitored free-living PA is closely related to total energy expenditure and captures both volitional and non-volitional activity.^28,29^ LTPA and walking for pleasure, on the other hand, can be considered volitional. Links between PA and well-being may operate directly through enhanced mood and indirectly through physical health benefits. The present hypothesis is that both free-living total PA and LTPA are positively associated with mental well-being. The aim of this study is to use data from a large, nationally representative cohort aged 60–64 years to investigate whether higher levels of monitored free-living PA and self-reported participation in LTPA are associated with higher levels of mental well-being, and to examine walking for pleasure specifically, the predominant activity reported for older adults.^30–32^

## Methods

### Study Sample

The Medical Research Council (MRC) National Survey of Health and Development (NSHD) is a nationally representative sample of all births in England, Scotland, and Wales during 1 week in March 1946. A random sample of these, stratified by father’s occupational class, has been followed on 23 occasions.^33,34^ The most recent data collection was in 2006–2011 (at age 60–64 years) and included assessment of PA and mental well-being. Of the original 5,362 study members at birth, 3,163 study participants known to be alive with an address in England, Scotland, or Wales were eligible to participate (excluding 718 who had died, 567 who were living abroad, 594 who had previously withdrawn from the study, and 320 who had been untraceable for >10 years).^35^ The sample is comparable with the 2001 English census with respect to several key socioeconomic and demographic characteristics.^35^ Of the 3,163 potentially eligible participants, 2,229 took part in a clinical assessment and were asked to participate in a free-living assessment of PA^33^ (Appendix Figure 1, available online).

The study received ethical approval from the Central Manchester Local Research Ethics Committee and the Scottish A Research Ethics Committee, and participants provided informed consent.

### Measures

Mental well-being was assessed using the Warwick-Edinburgh Mental Well-being Scale (WEMWBS),^36^ which was developed in 2006 to assess population mental well-being across a continuum given the increasing emphasis toward positive psychology. It includes items on positive affect, satisfying interpersonal relationships, and positive psychological functioning and has been validated in a representative general population sample of British adults.^36^ The scale has good content validity, high internal consistency (Cronbach’s α=0.91), and is unidimensional.^36^ Total scores were calculated by summing each participant’s responses on a 5-point Likert scale to each of 14 positively worded questions pertaining to the previous 14 days. If three or fewer items were missing (*n*=84 study participants), participant-specific means were imputed for each missing item; the one participant with more than three items missing was excluded. Possible total scores ranged between 14 and 70 with higher values indicating higher positive mental well-being.

A combined heart rate and acceleration monitor (Actiheart, CamNtech Ltd, Papworth, United Kingdom [UK]) was attached to the participants’ chest with two electrocardiogram electrodes. Data were collected at 30-second intervals for 5 consecutive days.^37^ Details of this assessment and derived data are provided elsewhere^38,39^ and summarized here. Heart rate data were individually calibrated,^40^ using data on response to an incremental aerobic exercise test (8-minute step test) conducted among eligible participants who attended a Clinical Research Facility (*n*=1,222; exclusion criteria for the step test included high blood pressure, history of unstable angina, or breathlessness). If the step test was not completed, group calibration was used. Data were adjusted for wear time and diurnal information bias and used in branched equation modeling^41^ to estimate intensity. This was summarized as total PA energy expenditure (PAEE; kJ/kg/day) and time spent in different intensities (based on a relative definition of 1 MET estimated using the Oxford equations for resting metabolic rate^42^: sedentary (≤1.5 METs), light (1.5–3 METs), and moderate to vigorous (>3 METs). After exclusion of participants with <48 hours of data and those with corrupt acceleration signals and/or severe clinical irregularities preventing valid heart rate measurement for extended periods (*n*=141), there were 1,787 participants with valid data on free-living activity (mean wear time, 4.9 days; SD=0.7 days).

Study members were also asked to complete a questionnaire that included the previously validated EPIC-Norfolk Physical Activity Questionnaire (EPAQ2),^43^ with minor modifications to include the addition of two “free text boxes” for participants to record other physical activities not in the predefined lists and questions on LTPA asked at previous data collection waves.^44^ LTPA was captured by asking how often in the previous 4 weeks study members had participated in any sports, vigorous leisure activities, or exercises, categorized as: no participation in LTPA, moderately active (one to four times), and most active (five or more times). Study members also reported their frequency and average duration of participation in walking for pleasure (not for transport) in the previous 12 months. This was categorized in hours per week as: none, >0 to ≤1, and >1.

Potential confounders were chosen a priori from known correlates of PA and mental well-being.^45–47^ These were gender, obesity, long-term limiting illness, smoking, work status, socioeconomic position (indicated by educational attainment and financial adequacy), personality traits (extraversion and neuroticism), and prior PA levels. Unless otherwise stated, these were assessed at age 60–64 years. Height and weight were measured and used to calculate BMI, which was categorized as not obese (<30 kg/m^2^) versus obese (≥30 kg/m^2^). The presence or absence of any long-term illness, health problem, or disability that limited activities was self-reported using a question from the 2001 England and Wales Census.^48^ Smoking was dichotomized as current versus ex/never smoker. Work status was categorized as not in paid work (mainly composed of those who were fully retired), working part-time, and working full-time. Highest level of educational attainment by age 26 years was categorized into three groups: no qualifications, lower level up to UK General Certificate of Education-Ordinary level (usually attained at age 16 years), and UK General Certificate of Education-Advanced level (usually attained at age 18 years) or higher. Financial adequacy at age 60–64 years was assessed by whether study members felt that with their present family income it was really quite hard to manage, possible to manage fairly well, or possible to manage comfortably. Extraversion and neuroticism were assessed at age 26 years using the Maudesley Personality Inventory^49^ and both scores were dichotomized as ≤6 versus 7–12.^50^ LTPA at age 53 years was assessed using the same questions as at age 60–64 years, and similarly categorized into three groups.

### Statistical Analysis

Associations between each potential confounder and WEMWBS score were examined in separate linear regression models. With the exception of obesity, each was statistically significant at the 5% level and was carried forward into subsequent analyses. Multiple linear regression models were used to test the relationships between each PA measure in turn and WEMWBS scores, adjusted for sex. No evidence of interaction between each PA measure and sex, or deviations from linearity for each PA measure, was found. Where there was evidence of association in sex-adjusted models, three additional models were run with sequential adjustments for (1) long-term limiting illness, smoking, educational attainment, financial adequacy, and work status; (2) extraversion and neuroticism; and (3) LTPA at 53 years. To reduce possible bias and loss of statistical power due to missing exposures and covariates (walking [0.2% missing], LTPA [<0.001%], long-term limiting illness [0.8%], financial adequacy [2.4%], educational level [5.4%], LTPA at age 53 years [5.3%], smoking [9.7%], and extraversion and neuroticism [10%]), these adjusted models were based on full information maximum likelihood (FIML) estimates^51,52^ using SEM (structural equation modeling) commands in Stata, version 12. Analyses were performed in 2013–2014.

### Sensitivity Analysis

All analyses were rerun on the sample with complete data on all covariates and effect estimates visually compared to those obtained from FIML estimation. To assess the reproducibility of findings on free-living PA, the following sex-adjusted analyses were run: (1) using time spent in different intensities of activity defined using a standard definition of 1 MET for all participants (i.e., 3.5 mL O_2_/kg/min [71.2 J/min/kg]) irrespective of individual differences in VO_2_max; (2) including only those individuals with individually calibrated PA estimates (*n*=881 among those who also had a valid WEMWBS score); and (3) using average trunk acceleration (m/s^2^) instead of PAEE (the latter estimated using data on both acceleration and heart rate).

## Results

A total of 1,976 study members (53% women, 47% men) of mean age 62.8 (SD=1.2) years had at least one measure of PA and a WEMWBS score. Men achieved slightly higher levels of PAEE and moderate to vigorous activity than women, whereas women were more likely than men to achieve light PA and report walking for pleasure for >1 hour/week (Table 1). Mean WEMWBS score was 51.6, consistent with population norms,^53^ and did not differ by sex.

In sex-adjusted analyses using the maximum available samples (Table 2), there was no evidence of associations between any of the free-living PA variables and WEMWBS scores, although associations were in the expected direction. A 1-SD increase in PAEE was associated with a 0.09 (95% CI= –0.32, 0.50) increase in WEMWBS. However, evidence suggested that participation in both LTPA and walking for pleasure were statistically associated with higher WEMWBS scores (Table 3). Those who participated in LTPA at least five times/month had mean WEMWBS scores 2.34 (95% CI=1.46, 3.23) points higher than those who reported no LTPA; those who walked for pleasure for >1 hour/week had mean WEMWBS scores 2.70 (95% CI=1.85, 3.55) points higher than those that reported no walking. The associations of both LTPA and walking for pleasure with WEMWBS scores were partially attenuated after adjustment for covariates but remained significant. However, in LTPA analyses, higher mean WEMWBS scores were only observed in those who reported participation in LTPA at least five times/month. Those who walked for pleasure for >0 to ≤1 or >1 hour/week had mean WEMWBS scores 1.58 (95% CI=0.61, 2.54) and 1.47 (95% CI=0.60, 2.34) points higher, respectively, compared with those that reported no walking after adjustment for all covariates.

The direction and magnitude of associations were similar when adjusted analyses were rerun on complete cases. There remained no evidence of associations between free-living PA variables and WEMWBS scores when using the standard definition of 1 MET for intensity estimates and including only individually calibrated data (Appendix Table 1, available online). However, an association between higher average trunk acceleration and higher WEMWBS scores was found; the mean sex-adjusted difference in WEMWBS score per 1-SD increase in acceleration was 0.64 (95% CI=0.23, 1.04).

## Discussion

In a large study of adults aged 60–64 years, participation in LTPA and walking for pleasure were associated with higher levels of mental well-being. Associations were robust to adjustment for sex, long-term limiting illness, educational attainment, financial adequacy, smoking, work status, personality, and prior LTPA. No evidence of associations between free-living PA measures and mental well-being was found.

The current results are consistent with evidence from other population-based studies that found modest cross-sectional associations between LTPA in older adults and better mental health.^22,54,55^ Furthermore, they agree with evidence from a study in older women, which found an association between more self-reported time spent walking and lower levels of anxiety and depression.^54^ The current results also complement the conclusions from two systematic reviews and meta-analyses of intervention studies in older adults.^18,19^ However, these other studies did not use an instrument designed to capture the multiple dimensions of positive mental functioning as well as affective elements of well-being, though other studies using WEMWBS are emerging.^56,57^

In contrast to NSHD findings, the two other studies that examined the relationship between free-living PA and indicators of improved mental well-being in older adults found some evidence of association, albeit weak.^26,27^ One possible reason for this discrepancy is that these other studies used accelerometry. Indeed, NSHD sensitivity analyses showed a positive association between average trunk acceleration and positive mental well-being. As accelerometers capture walking movements particularly well, this result is in line with the NSHD finding for self-reported walking.^58,59^ However, a strength of this current study is the use of combined heart rate and movement data, which are more closely associated with energy expenditure than estimates using movement alone.^28,29^ This suggests that higher levels of energy expenditure per se do not explain the associations of self-reported PA with well-being. Other possible reasons for the discrepancy in findings include the wider age ranges (i.e., 65 to ≥80 years^27^ and mean age around 75 [SD~4] years)^26^ and hence possible residual confounding by age. Additionally, in one study, participants were engaged in an intervention to encourage participation in regular, age-appropriate exercise.^26^

There are two main explanations for the finding that well-being was associated with participation in LTPA and walking for pleasure but not with free-living PA. First, the type and context of PA may be relevant for positive mental well-being. Free-living PA variables incorporate all incidental activities, some or all of which might be considered non-volitional and may include daily activities expected to have no or even a negative impact on mental well-being such as housework^60^ or transportation.^61^ LTPA is volitional; individuals choose the time, duration, and level that suit them.^25,62–64^ Although most intervention studies tested exercise of prescribed intensity and duration, there is evidence that exercise bouts that were self-selected or included an element of participant choice were more strongly related to improved mood.^19,20,62,65,66^ In addition, the items capturing LTPA and walking refer to the last 4 weeks and 12 months, respectively, and these timeframes may be more relevant for mental well-being than the 3–5 days over which free-living PA was assessed. Furthermore, walking is often cited as the most popular LTPA among older people^30^ and the benefits of this particular activity for mental well-being may be partly explained by its context (e.g., being undertaken in natural environments).^57,67^

An alternative explanation is that reporting bias underlies the associations, as LTPA, walking, and well-being were assessed by self-report. Adjustment for personality disposition (notably neuroticism) may go some way toward addressing this finding. Studies consistently find that people overestimate PA. Therefore, on the basis of these measures, this study does not make recommendations on the specific levels of activity that may promote well-being, but uses them to rank study members into those who undertook more or less activity.^68^

This study has numerous strengths. First, it uses data from combined heart rate and acceleration monitors to assess free-living PA and is complemented by self-reported PA collected at the same age.^28^ Second, it uses data from a large, nationally representative birth cohort and a validated scale designed to measure positive mental well-being. However, analyses were cross-sectional and the direction of associations could not be established. LTPA at age 53 years was associated with WEMWBS at age 60–64 years, but this did not fully explain the association of LTPA at age 60–64 years with WEMWBS. It seems likely that the relationship between PA and well-being across life is bi-directional. Additional limitations must also be acknowledged. In common with all longitudinal studies, there were losses to follow-up. Adjustment was made for health-related and socioeconomic factors that are associated with dropout, and FIML models were used to reduce the possibility of bias owing to missing covariates. In common with multiple imputation models, these are based on the untested assumptions that data are missing at random. Prior PA was controlled for as an important predictor of current PA,^38^ though only PA at age 53 years, and not more recently, was available. Neuroticism and extraversion, here captured at age 26 years, are recognized to be fairly stable but can change over a number of years.^69^

### Conclusions

Participation in LTPA and walking for pleasure are associated with mental well-being in adults aged 60–64 years. This suggests that promotion of participation in LTPA and walking for pleasure would not only provide known benefits for physical health and functioning but also have the associated benefit of better mental well-being.

## Figures and Tables

**Table 1:** Characteristics of the Medical Research Council National Survey of Health and Development^a^

	**Men**		**Women**		
	**N**^b^	**Mean (SD) or %**	**N**^b^	**Mean (SD) or %**	***p*****-value**^c^
WEMWBS score at 60–64 years	930	51.6 (7.9)	1046	51.6 (8.3)	0.9
Free-living physical activity at 60–64 years					
Physical activity energy expenditure (kJ/kg/day)	745	37.7 (15.7)	825	34.4 (13.2)	**<0.001**
Time spent in light physical activity (1.5–3.0 METs) (hours per day)	723	5.1 (1.6)	795	5.4 (1.5)	**<0.001**
Time spent in moderate to vigorous physical activity (>3 METs) (minutes per day)	723	73.7 (42.1, 119.3)^d^	795	68.8 (41.1, 108.6)^d^	**0.02**
Time spent sedentary (<1.5 METS) (hours per day)	723	17.4 (2.2)	795	17.3 (2.0)	0.17
LTPA at 60–64 years					
Participation in LTPA (no. times per month)					0.9
0	587	64.2	664	64.4	
1–4	128	14.0	136	13.2	
≥5	200	21.9	231	22.4	
Walking for pleasure (hours per week)					**<0.001**
None	304	32.7	273	26.1	
>0 to ≤1	262	28.2	279	26.7	
>1	364	39.1	493	47.2	
Covariates					
Obese at 60–64 years					
No	652	71.1	717	69.8	0.5
Yes	265	28.9	310	30.2	
Long-term limiting illness at 60–64 years					0.3
No	700	75.9	768	74.0	
Yes	222	24.1	270	26.0	
Educational attainment by 26 years					**<0.001**
No qualifications	278	31.7	311	31.3	
Lower level (up to O level or equivalent)^e^	181	20.7	354	35.7	
Higher level (A level and above)^f^	417	47.6	328	33.0	
Financial adequacy at 60–64 years					0.2
Quite hard to manage	67	7.4	61	6.0	
Manage fairly well	353	38.9	371	36.4	
Manage comfortably	488	53.7	588	57.7	
Smoking at 60–64 years					
Ex/Never	740	88.4	848	89.6	0.4
Current smoker	97	11.6	99	10.5	
Work status at 60–64 years					
Not in paid employment^g^	297	35.3	577	60.9	**<0.001**
Part-time paid employment	130	15.5	253	26.7	
Full-time paid employment	414	49.2	117	12.4	
Extraversion at 26 years					
Less	234	28.3	367	38.5	**<0.001**
More	592	71.7	587	61.5	
Neuroticism at 26 years					
Less	537	65.0	425	44.6	**<0.001**
More	289	35.0	528	55.4	
Self-reported participation in LTPA at 53 years (no. times per month)					0.1
0	365	42.4	462	45.7	
1–4	188	21.8	185	18.3	
≥ 5	308	35.8	364	36.0	

*Note:* Boldface indicates statistical significance (*p*<0.05).

a Medical Research Council National Survey of Health and Development (MRC NSHD). Sample restricted to those participants with data on WEMWBS and at least one physical activity measure at age 60–64 years (Maximum N=1,976).

b N varies due to missing data.

c *p*-values from chi-squared, t-test, or Wilcoxon rank-sum test of sex difference.

d Median (IQR).

e Lower level: up to UK General Certificate of Education-Ordinary level (usually attained at age 16 years).

f Higher level: UK General Certificate of Education-Advanced level (usually attained at age 18 years) or higher.

g % of this group who are fully retired: 95.3% men; 97.6% women.IQR, interquartile range; LTPA, leisure-time physical activity; WEMWBS, Warwick-Edinburgh Mental Wellbeing Scale.

**Table 2:** Sex-Adjusted Associations Between Free-Living Physical Activity and WEMWBS Scores at Age 60–64^a^

	**N**	**Mean difference in WEMWBS score**^b^**(95% CI)**	***p*****-value**
Physical activity energy expenditure (kJ/kg/day) per SD	1,570	0.09 (–0.32, 0.50)	0.7
Time per day spent in light physical activity (1.5–3.0 METs) per SD	1,518	0.05 (–0.36, 0.47)	0.8
Time per day spent in moderate to vigorous physical activity (>3 METs) per SD	1,518	0.15 (–0.26, 0.56)	0.5
Time per day spent sedentary (<1.5 METs) per SD	1,518	–0.11 (–0.52, 0.30)	0.6

a Data from the Medical Research Council National Survey of Health and Development.

b Differences in mean levels of WEMWBS score for a 1 SD increase in free-living physical activity estimated using multiple linear regression models.kg, kilogram; kJ, kilojoule; WEMWBS, Warwick-Edinburgh Mental Wellbeing Scale.

**Table 3:** Multiply Adjusted Associations Between Self-Reported Physical Activity and WEMWBS Scores at Age 60–64 (N=1,976)^a^

	**Mean difference in WEMWBS score**^b^**(95% CI)**			
	**Model 1**^c^	**Model 2**^d^	**Model 3**^e^	**Model 4**^f^
Participation in LTPA (no. times per month)				
0	0.00	0.00	0.00	0.00
1–4	1.90 (0.83, 2.97)	1.18 (0.19, 2.24)	0.95 (−0.10, 2.00)	0.82 (−0.25, 1.88)
≥5	2.34 (1.46, 3.23)	1.67 (0.79, 2.55)	1.56 (0.68, 2.43)	1.25 (0.34, 2.16)
	***p*****<0.001**	***p*****<0.001**	***p*****=0.001**	***p*****=0.02**
Walking for pleasure (hours per week)				
None	0.00	0.00	0.00	0.00
>0 to ≤1	2.77 (1.83, 3.71)	1.75 (0.77, 2.72)	1.69 (0.73, 2.65)	1.58 (0.61, 2.54)
>1	2.70 (1.85, 3.55)	1.86 (0.99, 2.73)	1.67 (0.81, 2.53)	1.47 (0.60, 2.34)
	***p*****<0.001**	***p*****<0.001**	***p*****<0.001**	***p*****=0.001**

*Note:* Boldface indicates statistical significance (*p*<0.05).

a Data from the Medical Research Council National Survey of Health and Development.

b Differences in mean levels of WEMWBS score by categories of self-reported physical activity estimated using multiple linear regression models.

c Model 1: Adjusted for sex.

d Model 2: Model 1 plus long-term limiting illness, educational attainment by 26 years, financial adequacy at 60–64 years, smoking at 60–64 years, and work status at 60–64 years.

e Model 3: Model 2 plus extraversion and neuroticism at 26 years.

f Model 4: Model 3 plus LTPA at 53 years.LTPA, leisure-time physical activity; WEMWBS, Warwick-Edinburgh Mental Wellbeing Scale.

## Footnotes

Appendix Supplementary data associated with this article can be found at, doi:10.1016/j.amepre.2015.03.009.
