# Supplementary material for: Physical Activity and Mental Well-being in a Cohort Aged 60–64 Years
Source: Am J Prev Med. 2015 Aug;49(2):172–80. doi: 10.1016/j.amepre.2015.03.009 (PMC4518501; doi:10.1016/j.amepre.2015.03.009)

Appendix Table 1. Sensitivity Analyses: Sex-Adjusted Associations Between Alternative Free-Living Physical Activity Measures and WEMWBS at Age 60-64[a]

		N	Mean difference in WEMWBS[c] (95% CI)
i)	**Intensity of activity defined using 1 standard MET[b]**		
	Time per day spent in light to moderate physical activity (1.5 - 3.0 METs) per SD	1,518	-0.06 (-0.48, 0.35) *p*=0.76
	Time per day spent in moderate to vigorous physical activity (> 3.0 METs) per SD	1,518	0.14 (-0.29, 0.56) *p*=0.5
	Time per day spent sedentary (<1.5 METs) per SD	1,518	0.01 (-0.40, 0.42) *p*=0.95
ii)	**Sample restricted to those with individually calibrated data**		
	Physical activity energy expenditure (kj/kg/day) per SD	881	-0.02 (-0.05, 0.02) *p*=0.34
iii)	**Average trunk acceleration (m/s^2) per SD**	1,515	**0.64 (0.23, 1.04) *p*=0.002**

Note: Boldface indicates statistical significance ($p<0.05$).
WEMWBS, Warwick-Edinburgh Mental Wellbeing Scale; MET, metabolic equivalent
[a] Data from the MRC National Survey of Health and Development
[b] 3.5 ml O_2/kg/min (71.2 J/min/kg)
[c] Differences in mean levels of WEMWBS score for a 1SD increase in free-living physical activity estimated using multiple linear regression models

Appendix Figure 1. Flowchart summarizing participation in the MRC National Survey of Health and Development at age 60-64 years (in 2006-2011)

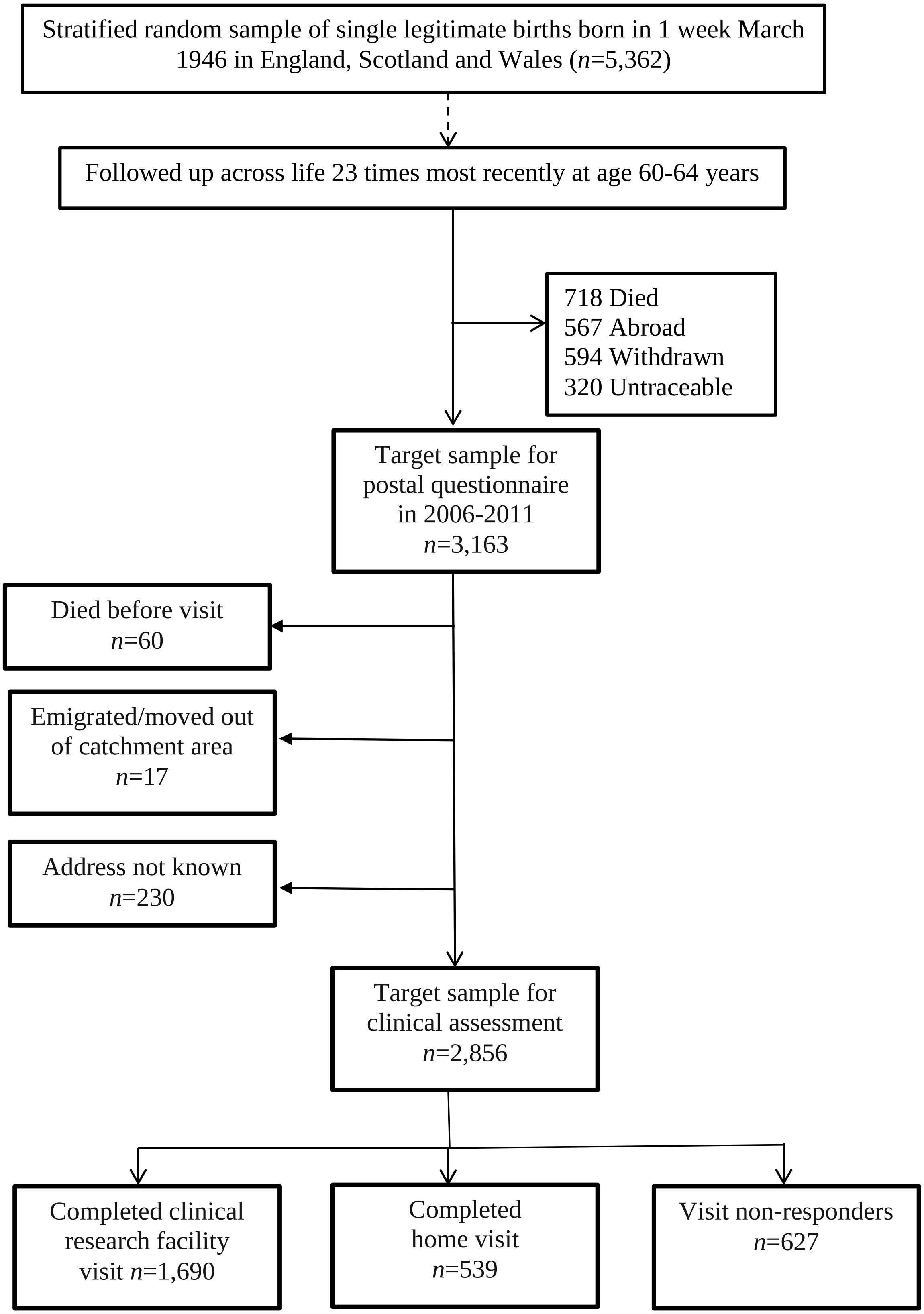

Supplement: Supplementary file 1 — Supplementary Material [file mmc1.pdf]
